# Management of chyle leaks following esophageal resection: a systematic review

**DOI:** 10.1093/dote/doab012

**Published:** 2021-03-16

**Authors:** Robert Power, Philip Smyth, Noel E Donlon, Timothy Nugent, Claire L Donohoe, John V Reynolds

**Affiliations:** National Oesophageal and Gastric Centre, St James's Hospital, Dublin, Ireland; National Oesophageal and Gastric Centre, St James's Hospital, Dublin, Ireland; National Oesophageal and Gastric Centre, St James's Hospital, Dublin, Ireland; National Oesophageal and Gastric Centre, St James's Hospital, Dublin, Ireland; National Oesophageal and Gastric Centre, St James's Hospital, Dublin, Ireland; National Oesophageal and Gastric Centre, St James's Hospital, Dublin, Ireland

**Keywords:** chylothorax, esophageal adenocarcinoma, esophageal and gastric surgery, esophagectomy, esophagogastric surgery

## Abstract

**Background:**

Chyle leakage is an uncommon but potentially life-threatening complication following esophageal resections. The optimal management strategy is not clear, with a limited evidence base.

**Methods:**

Searches were conducted up to 31 December 2020 on MEDLINE, Embase, and Web of Science for randomized trials or retrospective studies that evaluated the management of chyle leakage following esophageal resection. Two authors independently screened studies, extracted data, and assessed for bias. The protocol was prospectively registered on PROSPERO (CRD: 42021224895) and reported in accordance with preferred reporting items for systematic reviews and meta-analyses guidelines.

**Results:**

A total of 530 citations were reviewed. Twenty-five studies, totaling 1016 patients met the inclusion criteria, including two low-quality clinical trials and 23 retrospective case series. Heterogeneity of study design and outcomes prevented meta-analysis. The overall incidence of chyle leak/fistula was 3.2%. Eighteen studies describe management of chyle leaks conservatively, 17 by surgical ligation of the thoracic duct, 5 by pleurodesis, and 6 described percutaneous lymphangiography with thoracic duct embolization or disruption.

**Conclusions:**

The evidence base for optimal management of chyle leakage postesophagectomy is lacking, which may be related to its low incidence. There is a paucity of high-quality prospective studies directly comparing treatment modalities, but there is some low-certainty evidence that percutaneous approaches have reduced morbidity but lower efficacy compared with surgery. Further high-quality, prospective studies that compare interventions at different levels of severity are needed to determine the optimal approach to treatment.

## INTRODUCTION

Chyle leakage from major lymphatic channels, and resulting in a chylothorax or fistula, is an uncommon but potentially life-threatening complication of esophageal resectional surgery, in particular for esophageal cancer.^1^^,^^2^ Most commonly, this relates to the thoracic phase of esophagectomy, where the thoracic duct is injured, or excised *en bloc* with proximal clipping or ligating which fails, or due to injury of unidentified tributaries or anatomic variations such as a double thoracic duct.^3–5^ It may also relate to the abdominal dissection, with iatrogenic injury to the cisterna chyli, which can lead to chylous ascites as well as chylothorax.^6^ The thoracic duct has a rate of flow of up to 4 L/day and contains lymph that is rich in fats, fat-soluble vitamins, proteins, and lymphocytes.^7^ The clinical consequences accordingly may be substantial from large volume leakage, with hypoalbuminemia and lymphopenia resulting in malnutrition and immunosuppression and increased risk of infection and sepsis.^8^

The most common clinical presentation in the postoperative period is with excessive external drainage via a chest thoracostomy in the early postoperative period or a large effusion (chylothorax) where no drain is present. Since enteral nutrition via a feeding jejunostomy or nasojejunally is commonly begun on the first postoperative day, the drainage of cream-like effluent is the most common observation that establishes a clinical diagnosis.^9^ This may be confirmed with laboratory evidence of a triglyceride levels >110 mg/dL (6.1 mmol/L) or less commonly via the confirmation of chylomicrons in the effluent. The volume of drainage is important, particularly if over 1 L in 24 hours that may suggest complete disruption of the thoracic duct. The response to discontinuation of enteral feeding also has prognostic significance, particularly if it does not impact on the volume of drainage.^10^

In the most common scenario the initial approach to management is conservative, including external drainage, dietary modifications like total parental nutrition (TPN) or enteral medium chain triglycerides (MCT), and somatostatin analogues.^11^ Reoperation and surgical thoracic duct ligation (TDL) have traditionally been reserved for cases refractory to conservative treatment, illustrated by persistently high output or a deteriorating clinical picture.^12^ In recent years, interventional radiology (IR) approaches have emerged as a potentially less-invasive alternative with thoracic duct embolization (TDE) using coils or embolic agents.^13^^,^^14^ If TDE fails, thoracic duct disruption (TDD) can interrupt lymphatic flow by needle punctures at the level of the cisterna chylii.^15^

Although the consequences of chyle leakage are well recognized, there is no international consensus on the best approach to management of severe or refractory cases. In particular, questions remain regarding the timing of and criteria for invasive interventions as well as the effectiveness of IR approaches and a clear overall treatment algorithm. To this end, we conducted a systematic literature search with the goal of synthesizing the evidence base and informing a discussion that may result in clear guidelines.

## METHODS

This study was conducted in accordance with the preferred reporting items for systematic reviews and meta-analyses (PRISMA).^16^ The PRISMA checklist can be found in the Supplementary Table S1. The protocol for this systematic review was prospectively registered on PROSPERO (CRD: 42021224895).

### Search strategy

A search was carried out on MEDLINE (PubMed), Embase, and Web of Science on 31 December 2020. The strategy included medical subject heading, free text words and synonyms covering ‘chylothorax’, ‘chyle leak’, ‘esophagectomy’ and was restricted to studies in English and conducted in humans. The full search strategy is available in the supplementary material. All references from relevant systematic reviews were hand searched for additional studies, and all duplicate records were removed using the Covidence Systematic Review Manager.

### Screening and article selection

**Fig. 1:**
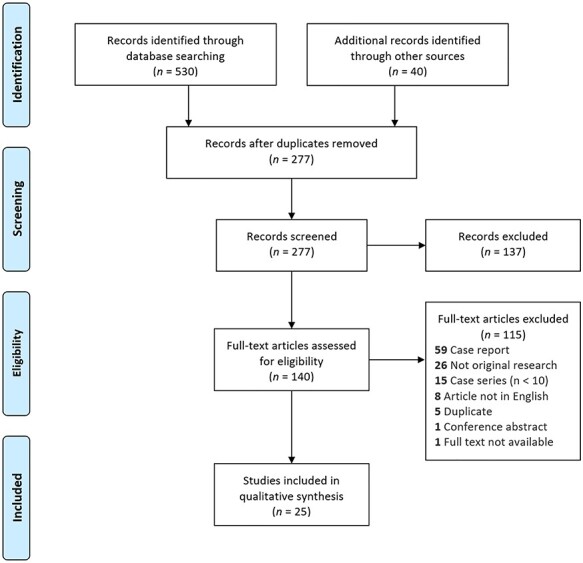
PRISMA flow diagram of the search process.

Two independent authors (R.P. and T.N.) reviewed the titles and abstracts to identify relevant studies. Full-text manuscripts were then assessed independently by two reviewers (R.P. and T.N.) against predefined inclusion criteria using the population, intervention, comparator, outcomes, and study type (PICOS) framework. The population of interest was patients with a chyle leak after esophagectomy. The interventions and comparators were conservative management, surgical TDL, TDE, and TDD. The outcomes were technical success rate, clinical success rate, time to resolution of chyle leak, complications of procedures, and the study types were retrospective case series and clinical trials. Studies that included chyle leaks of multiple etiologies were included as long as there were at least 10 relevant cases. Case reports, small case series (*n* < 10), conference abstracts, commentaries, editorials were excluded. Studies that only reported the incidence, risk factors, or prognosis but not management were excluded. Any disagreement between reviewers was resolved by discussion until consensus was reached. The study selection procedure is presented using a PRISMA flowchart (Fig. 1).

### Data extraction and evidence synthesis

Two authors (R.P. and P.S.) independently extracted data, conflicts were resolved by discussion, and findings were reported in accordance with PRISMA guidelines. Data were extracted using the Covidence Systematic Review Manager in a standardized proforma under the following headings: study ID, country, study design, study type, study period, inclusion criteria, total number of patients, prevalence of chyle leakage, risk factors, management strategies, technical success rates, clinical success rates, complications, and impact on prognosis.

Meta-analysis of the data was not possible due to heterogeneity in subjects, study design, management strategies, and clinical endpoints. Therefore, the extracted data are presented as a qualitative synthesis.

### Quality assessment

The risk of bias in included randomized controlled trials (RCTs) was assessed using Cochrane’s Risk of bias tool.^17^ Quality assessment of retrospective case series was done using the National Institute of Heart Lung and Blood (NHLBI) quality assessment tool for nonrandomized case series.^18^

## RESULTS

### Characteristics of included studies

The original search identified 530 studies and a further 40 studies were added from the references of another systematic review (Fig. 1). After duplicates were removed, 277 studies remained with 137 of these excluded following title and abstract screening. Of the 140 that were reviewed by full text, 25 studies matched all the eligibility criteria^10^^,^^19–42^. The overall incidence rate was 3.2% (784/24 510, 18 studies) with a range of 1.1–21%. No consistent risk factors for chyle leakage were reported across studies. Two studies were clinical trials,^19^^,^^28^ whereas the other 23 were retrospective case series. Four studies did not outline their definition of a chyle leak.^19^^,^^31^^,^^34^^,^^36^ One study used the Esophageal Complications Consensus Group (ECCG) definition of chyle leaks,^39^ in 4 studies this was a clinical diagnosis based on quantity and quality of chest drainage,^21–23^^,^^42^ and the remaining 16 confirmed this clinical diagnosis biochemically with triglycerides and/or chylomicrons in chest drain fluid. A summary of study characteristics is presented in Table 1.

**Table 1:** Characteristics of included studies

**Study**	**Country**	**Study design**	**Period**	**Inclusion criteria**	***N***	**Incidence (%)**
Alamdari *et al*. 2018^19^	Iran	Clinical trial	2009–2014	Postesophagectomy	52	—
Alexiou *et al*. 1998^20^	United Kingdom	Retrospective case series	1987–1997	Postesophagectomy	21	4
Boffa *et al*.2008^21^	United States	Retrospective case series	2003–2006	Multiple etiologies	26	—
Bolger *et al.* 1991^22^	Ireland	Retrospective case series	1977–1990	Postesophagectomy	11	2
Brinkmann *et al.* 2016^23^	Germany	Retrospective case series	2005–2013	Postesophagectomy	17	1.9
Dugue *et al.* 1998^10^	France	Retrospective case series	1980–1996	Postesophagectomy	23	2.7
Fujita and Daiko 2014^41^	Japan	Retrospective case series	2001–2009	Postesophagectomy	20	3.8
Itkin *et al.* 2010^24^	United States	Retrospective case series	1996–2009	Multiple etiologies	31	—
Kim *et al.* 2014^25^	South Korea	Retrospective case series	1994–2010	Postesophagectomy	57	3.8
Kranzfelder *et al.* 2013^26^	Germany	Retrospective case series	—	Postesophagectomy	39	2.1
Lagarde *et al.* 2005^27^	The Netherlands	Retrospective case series	1995–2003	Postesophagectomy	20	3.7
Li *et al*. 2013^28^	China	Clinical trial	1996–2011	Postesophagectomy	306	2.9
Merigliano *et al*. 2000^29^	Italy	Retrospective case series	1980–1998	Postesophagectomy	19	1.1
Miao *et al*. 2015^30^	China	Retrospective case series	2007–2012	Postesophagectomy	34	2.6
Milito *et al*. 2020^42^	United Kingdom	Retrospective case series	1997–2017	Postesophagectomy	50	5
Nadolski and Itkin 2018^31^	United States	Retrospective case series	2003–2016	Multiple etiologies	13	—
Ohkura *et al*. 2018^32^	Japan	Retrospective case series	2011–2017	Postesophagectomy	19	5.1
Pamarthi *et al*. 2014^33^	United States	Retrospective case series	2002–2011	Multiple etiologies	43	—
Paul *et al*. 2009^34^	United States	Retrospective case series	1992–2008	Multiple etiologies	12	2.6
Rao *et al*. 2004^35^	India	Retrospective case series	1982–2002	Postesophagectomy	14	2.5
Reisenauer *et al*. 2018^36^	United States	Retrospective case series	2008–2015	Multiple etiologies	46	—
Schumacher *et al*. 2007^37^	Germany	Retrospective case series	1988–2005	Postesophagectomy	10	2.4
Shah *et al*. 2012^38^	United States	Retrospective case series	1997–2008	Postesophagectomy	34	3.8
Weijs *et al*. 2017^39^	The Netherlands	Retrospective case series	2003–2014	Postesophagectomy	78	21
Yannes *et al*. 2017^40^	United States	Retrospective case series	2011–2015	Multiple etiologies	21	—

The two clinical trials were found to have a high risk of bias,^19^^,^^28^ using the Cochrane Risk of Bias 2.0 tool. Using the NHLBI quality assessment tool, 1 study had an overall assessment of poor quality,^34^ 9 were fair,^10^^,^^21^^,^^26^^,^^30–32^^,^^35^^,^^36^ and the remaining 13 were good quality. A detailed breakdown of the quality assessment for the clinical trials and observational studies can be found in Supplementary Figure S1 and Supplementary Table S2, respectively.

### Conservative management

Sixteen studies, including a total of 762 patients, reported outcomes of a conservative management regimen (Table 2). Most regimens consisted of TPN and/or enteral dietary modification with continued chest drainage.^10^^,^^20^^,^^22^^,^^23^^,^^25–30^^,^^32^^,^^35^^,^^38^^,^^39^^,^^41^^,^^42^ Enteral MCT nutrition were used in three studies; in 1 instead of TPN,^26^ in another before TPN,^42^ and the other did not report the sequence of nutritional modification.^38^ One series reported the use of elemental low-fat tube feeding if chyle output was <500 mL/24 hours, and TPN if this output exceeded this threshold.^39^ Clinical success rates ranged from 36.3% to 86.6%. Seven studies included the use of octreotide, a somatostatin analogue yielding clinical success rates ranging from 38% to 100%. One retrospective case series compared the effectiveness of adding octreotide with conventional treatment, consisting of chest drainage, TPN with or without pleurodesis, after thoracic esophagectomy, and had a higher success rate (86.6% vs. 40%, *P* = 0.03) with no adverse events.^41^ Etilefrine, an α-and β-adrenergic agonist that causes contraction of thoracic duct smooth muscle, was described in one case series as an adjunct to TPN, chest drainage, and pleurodesis.^32^ There was a trend toward a longer duration of chylothorax in the no-etilefrine group (*n* = 5) than in the etilefrine group (*n* = 11) (27.8 vs. 11.6 days; *P* = 0.078). There was no improvement in clinical success rate with 75% achieving a cure in the etilefrine group versus 100% in the no-etilefrine group.

**Table 2:** Conservative management of chyle leaks

**Study**	**Conservative approach**		**Clinical success (%)**	**Mortality (%)**	**Other outcomes**
Alexiou *et al.* 1998^20^	TPN and chest drain		64.7	23.5	Mean TTR 14.7 days
Bolger *et al.* 1991^22^	TPN and chest drain		72	36.3	Median TTR 35 days
Brinkmann *et al.* 2016^23^	TPN ± additional pleural drainage		11	11.8	Median LOS 30 days
Dugue *et al.* 1998^10^	TPN and chest drain		61	0	Mean delay of 12 days
Kim *et al.* 2014^25^	TPN, chest drain ± octreotide ± pleurodesis		75.4	—	—
Fujita and Daiko 2014^41^	TPN, chest drain and octreotide		86.6	—	Mean TTR 5 days
	TPN, chest drain		40	—	Mean LOS 36 days
Kranzfelder *et al.* 2013^26^	MCT, rehydration, and chest drain		83	16	—
Lagarde *et al.* 2005^27^	TPN and chest drain		80	0	—
Li *et al*. 2013^28^	48 hours of	Hydration, IV supplementation of albumin and/or plasma to maintain total protein levels >70 g/L and albumin levels >35 g/L, and chest drain	37.8	26	—
	2 weeks of		70	5	—
Merigliano *et al*. 2000^29^	TPN and chest drain		36.3	0	Median TTR 14 days
Miao *et al*. 2015^30^	TPN, chest drain, and octreotide		68	2.9	Median TTR 12 days
Milito *et al*. 2020^42^	Fluid and electrolyte maintenance, MCT or ‘rarely’ TPN, chest drain and co-trimoxazole if lymphocyte count was <1000/μL		48	8.3	—
Ohkura *et al*. 2018^32^	TPN, octreotide ± pleurodesis with OK-432 (effluent <50–100 mL/day)		100	0	—
	TPN, octreotide, etilefrine ± pleurodesis with OK-432 (effluent <50–100 mL/day)		75	—	—
Rao *et al*. 2004^35^	IV fluid and albumin supplementation, TPN, chest drain ± octreotide		50	28.6	Median LOS of 20 day
Shah *et al*. 2012^38^	MCT (52%), TPN (63%), chest drain (44%) ± octreotide (31%) ± pleurodesis (16%)		38	—	—
Weijs *et al*. 2017^39^	Elemental low-fat tube feeding (if output <500 mL/24 hours), TPN (If output<500–1000 mL/24 hours)		66	2	Median TTR 9 days

Abbreviations: TTR, time to response; LOS, length of stay in hospital.

**Table 3:** Interventional management of chyle leaks

**Study**	**Patient selection**	**Intervention**	***N***	**Technical success (%)**	**Clinical success (%)**	**Other outcomes**
Boffa *et al.* 2008^21^	Unknown indication/ referrals	TDE	21	93	57	Median time to discharge 8 days
		TDD	4	—	50	Median time to discharge 19 days
Itkin *et al.* 2010^24^	Unknown indication/referrals	TDE	73	97	74.6	—
		TDD	18	—	72	—
Nadolski and Itkin 2018^31^	Failed TDL/referral	TDE	49	98	98	—
		TDD	1	100	100	—
Pamarthi *et al*. 2014^33^	Unknown indication/referrals	TDE/TDD	50	86	56	—
Reisenauer *et al*. 2018^36^	1.1 L daily output	Surgical TDL	48	—	85	—
		TDE	40	48	38	8% mortality, 50% clinical success postesophagectomy (*n* = 22)
Yannes *et al*. 2017^40^	Failed medical conservative in confirmed chyle leak postop	INL alone	7	100	71.4	3% (1) mortality; median TRR 14 days
		INL + TDE	21	—	90.5	Median TTR 3 days
		INL + TDD	12	—	41.7	Median TTR 7 days

### Surgical management

Seventeen studies included surgical TDL in the management of chyle leaks, reporting on a total of 767 patients.^10^^,^^19^^,^^22^^,^^23^^,^^26^^,^^28–30^^,^^34–39^^,^^43^^,^^44^ Eleven of these retrospectively compared patients who received conservative management with those who initially underwent conservative management that failed prior to subsequent TDL. The time to reoperation in the latter subgroup was variable, ranging from 5 to 39 days and the rate of reoperation ranged from 23.5% to 88.2%. In a majority of studies, the indication for reoperation was vague and limited to persistent or increasing chyle leak and/or hemodynamic instability.

Seven studies included more explicit indications. These varied between a chest drain output exceeding a defined volume, ranging from 100 mL to 1.1 L per day and/or a chyle leak persistent after 2 to 14 days of conservative management.^19^^,^^25^^,^^26^^,^^28^^,^^36^^,^^39^^,^^42^^,^^44^ Miao *et al.*^30^ and Shah *et al.*^38^ retrospectively identified a common flow rate threshold for patients undergoing reoperation, which stood at 13.5 mL/kg per day and 11.6 mL/kg per day, respectively. Clinical success in the conservatively managed group ranged from 11% to 75.4%. In all but four studies,^30^^,^^42^ patients who failed conservative management were also included in the reoperation subgroup in which clinical success rates ranged from 31% to 100% with a technical success rate, defined by successful identification and ligation of the leaking duct, of 100% in six out of the seven studies that reported it. Mortality after TDL ranged from 0% to 33%. Minor complications were reported in four studies and included lung parenchymal injury, pneumonia (28.5–33%), wound infection or dehiscence (21.4%), and prolonged thoracostomy drainage (6.6%).

Two studies compared early and delayed reoperation. One nonrandomized clinical trial (*n* = 306), with a high risk of bias, assigned patients to TDL after 48 hours or after 14 days of failed conservative management as defined by daily chyle output over 1 L per day at the respective time points.^28^ The overall rates of morbidity (early: 31.7% vs. late: 15%, *P* = 0.001) and mortality (early: 14% vs. late: 4.2%, *P* = 0.006) were higher in patients who underwent early TDL. The authors attributed this difference to the higher rate of reoperation in the aggressively managed cohort (early: 72.6% vs. late: 30%). Technical success rates were not reported for either group. The other study was a retrospective case series that compared patients who underwent conservative management and reoperation after 48 hours and patients who underwent conservative management and delayed reoperation at a median of 12 days.^29^ The major finding was a significantly shortened hospital stay in the aggressively managed group (median 20.6 vs. 37 days, *P* = 0.007) and no differences in overall mortality between both treatment arms.

### Pleurodesis

Three studies included pleurodesis as a component of conservative management.^25^^,^^32^^,^^38^ Ohkura *et al.*^32^ carried out pleurodesis with picibanil (OK-432, a sclerosant) following TPN and either octreotide or etilefrine, if the effluent was <50–100 mL/day. This was unsuccessful in all five patients treated. The remaining two studies did not describe the indications or method of pleurodesis, and outcomes were not reported separately.^25^^,^^38^ Two studies, including one RCT, compared TDL to pleurodesis. The trial, which had a high risk of bias, randomized 52 patients who did not respond to conservative management to either surgical TDL or pleurodesis using platelet-rich fibrin glue.^19^ Clinical success, defined as daily chylous drainage <100 mL/day, was higher in the pleurodesis group (100% vs. 77% *P* = 0.009). There was also an associated significantly shorter overall hospital and intensive care unit length of stay (53 vs. 36 days, *P* < 0.05) in the pleurodesis group, and a trend toward reduced mortality (15% vs. 3.74%, *P* = 0.1612). The other study was a retrospective case series (*n* = 12) reporting a numerically higher clinical success rate with TDL in comparison to talc pleurodesis (95% vs. 83%, no significance reported).^34^

### IR approaches

Six retrospective case series described the use of percutaneous IR intervention (Table 1). In total, 455 patients with chyle leaks from multiple etiologies were studied, including 180 postesophagectomy patients. Of the six studies, two further stratified success rates for postesophagectomy patients. Pamarthi et *al.*^33^ reported a technical success rate of 86% for this patient subgroup (*n* = 43), and an overall clinical success rate of 56%. Yannes et *al.*^40^ reported clinical success rates of 88.6% for TDE (*n* = 9), and 75% in TDD (*n* = 4) following esophagectomy. Notably, the odds of clinical success of TDE/TDD in treating postsurgical chylothorax did not differ based on the type of operation (*P* = 0.67). In the remaining studies, the median technical success rate for percutaneous intervention was 93% (range: 48–100%) and the median clinical success rate was 57% (range: 38–98%). Four studies reported the outcome of TDE and TDD separately, with a median clinical success rate for TDE of 74.6% (range: 57–98%) and a median clinical success rate for TDD of 72% (range: 41.7–100%).^21^^,^^24^^,^^31^^,^^40^ The rate of minor complications ranged from 4% to 6% and included further chyle leak, leg and pedal edema, asymptomatic pulmonary embolization, and inconsequential coil misplacement. A mortality rate was present in only two studies and stood at 3% (*n* = 1)^40^ and 8% (*n* = 1)^36^ with the latter death occurring after discharge. Additionally, TDE was associated with a shorter time to resolution (3 vs. 7 days; *P* = 0.007)^40^ and a shorter hospital stay (8 vs. 19 days)^21^ in comparison to TDD. In another retrospective study (*n* = 21), patients were assigned to intranodal lymphangiography (ILN) alone, INL and TDE, or INL and TDD according to daily chyle output and the presence/absence of leak on INL. At a nonspecified time after the diagnosis of a chyle leak, patients with drainage below 500 mL/day only received TDE/TDD if a leak was identified on INL, whereas all patients with drainage above 500 mL/day received TDE/TDD. Interestingly, there was no difference in clinical success rates between INL alone and INL and TDE/TDD when controlling for chyle output (*P* = 0.19).^40^ In the retrospective case series comparing surgical TDL to TDE (*n* = 46), clinical success rates were superior in the TDL group that in the TDE group (85% vs. 38%) with a major contributing factor to poor success rate with TDE being an inability to cannulate the cisterna chyli in 48% of patients.^36^

## DISCUSSION

This systematic review clearly highlights the lack of existing high-quality studies to inform optimal management of chyle leaks after esophageal resection. The strength of evidence is low, and most studies are retrospective. For the few reported trials, a significant risk of bias is evident. The literature is further complicated by a lack of consensus up to recently on definitions, the heterogeneity of management regimens used, and the variability of treatment modalities composing these regimens.

In this context, a major advance in classification of chyle leaks postesophagectomy was the recent agreed definition based on Delphi Consensus of the ECCG, a collaborative group of 24 high-volume surgical centers across 14 countries. In this schema, chyle leaks were classified based on response to treatment: type I requiring enteral dietary modification; type II requiring TPN; and type III requiring interventional or surgical treatment.^44^ Further division is possible based on output volume: type A with <1 L daily output and type B with >1 L daily output.^45^

**Fig. 2:**
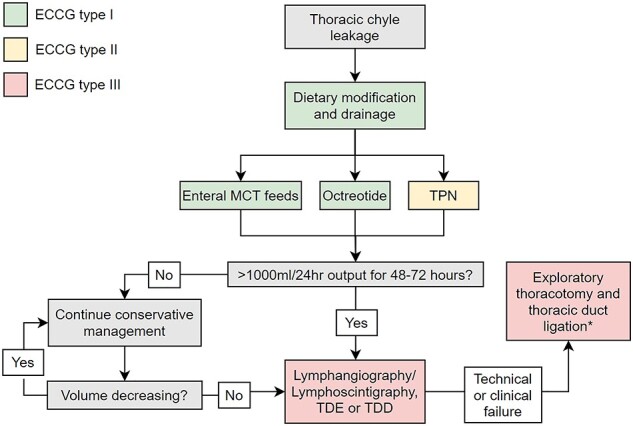
Summary of management of chyle leakage following esophagectomy. The ECCG has classified chyle leaks based on treatment: type I requiring enteral dietary modification like MCT; type II requiring TPN; and type III requiring surgical treatment, or IR-based approaches including TDE or TDD.^44^^*^Pleurodesis is a potential addition or alternative to surgical TDL, but more evidence is needed before it can be recommended routinely.

The recent nomenclature and classification arising out of the ECCG consensus will be helpful in addressing some of these gaps in future studies. Applying this retrospectively, although inexact, is consistent with an approach that conservative management has been the mainstay of management for type I and II leaks, with good success rates (Fig. 2). Stopping standard enteral nutrition and using TPN or enteral MCTs would be a standard initial approach. What is unclear is whether TPN or enteral MCTs are equal alternatives or sequential considerations if TPN and enteral rest have achieved a target reduction in output. The evidence for octreotide largely comes from managing chyle leakage of other etiologies, including idiopathic and postsurgical chylothoraces, and most studies report this to be a safe and modestly effective approach, but it remains unclear as to its specific benefit in the context of esophageal resectional surgery and whether the type of leak has relevance.^46–48^ Etilefrine, a sympathomimetic agent, is under investigation as another component of conservative management, mostly based on experience in other etiologies.^49–51^ However, the sole study evaluating its use in the postesophagectomy context did not demonstrate significant efficacy, ^32^ and there is insufficient evidence to recommend it for routine use.

For complicated fistulae, type III by the ECCG terminology, and where conservative measures have not been successful, reoperation and surgical ligation of the thoracic duct (TDL) has been the traditional gold standard.^11^ This can be carried out by open thoracotomy or through video-assisted thoracoscopy.^12^ In 16 studies that described this strategy, TDL had a high success rate, with rates of resolution in excess of 80%. The key questions relate to indications and timing, and whether the advent of interventional radiological TDE/TDD approaches are better initial options. Indications for TDL are often poorly described and may vary across studies. They were reported in seven studies, in some it was based on daily measured chyle output, in others based on refractoriness to a conservative strategy, strongly suggesting that this decision in published studies has been predominantly based on surgeon’s preference and subjective factors in addition to objective findings. Optimal timing is also unclear, a low-quality nonrandomized trial included found higher morbidity and mortality in early reoperation, at 48 hours, cautioning against this approach. Conversely, a retrospective series found that early reoperation was associated with shortened hospital stay. Pleurodesis is rarely used in Europe^42^; however, one low-quality randomized trial in our search compared it with surgical TDL and found a higher rate of clinical success with pleurodesis, clearly rigorous trials are required.^19^ An important consideration, outside the scope of this review, is whether routine identification of the thoracic duct at surgery, its excision, and whether ligation is one by clips, or hem-o-lock, or sutures, impacts on both the incidence of chyle leakage, its severity, and the optimal management approach.

IR approaches via TDE and TDD has provided an important new dimension to the diagnosis and management of chyle leaks, in particular the most complex cases. First described by Cope in 1998,^13^ lymphangiography, TDE, and TDD now present major interventional options.^14^ A major advantage is the low complication rate, as a meta-analysis of lymphatic interventions for chylothorax found that the pooled major complication rate of TDE and TDD were 1.9% (95% confidence interval [CI], 0.8–4.3%) and 2.4% (95% CI, 0.9–6.6%), respectively.^43^ In this review, just two deaths were reported in the 358 patients that underwent TDE and TDD in six studies, and complications were rare and minor in severity. The major caveat relates to inferior efficacies that relates in large part to technical expertise. Highlighting this is one recent case series that compared chyle leakage patients managed by either TDL or TDE found that rates of clinical success were 85% and 38%, respectively. Conversely, in a meta-analysis including chyle leakage of myriad etiologies, the pooled clinical success rates of TDE was 79.4% (95% CI, 64.8%–89.0%), and that of TDD was 60.8% (95% CI, 49.4%–71.2%).^43^ High-quality prospective studies that directly compare TDL and embolization or disruption of the thoracic duct for the management of type III chyle leak are needed to definitively answer this clinical question. Notwithstanding, the current digest of the literature would suggest that IR with a view to diagnosis and treatment may be the first step in type III fistulae and TDL reserved for where this fails (Fig. 2).

This is the largest systematic review of management of chyle leaks following esophageal resections. Clear conclusions are limited by the lack of clear terminology across studies as well as consistent indications for intervention, the heterogeneity of patient cohorts, and the retrospective gathering of data. The review however provides a summary of the literature, and its quality, and informs a starting point, enabled by the ECCG classification and such international network, to explore the management of chyle leakage in rigorous prospective study within the Esodata Registry. This can form the basis for a higher-quality observational data, which could inform more robust clinical recommendations. Several important clinical questions could benefit from such multicenter collaboration, include the role of IR approaches, the timing of interventions for type III leaks, the duration of conservative management in complex leaks, and what constitutes optimum conservative management, including the sequencing of TPN and MCT, and the role of octreotide and pleurodesis play.

In conclusion, the grade of evidence for optimum management of chyle leakage postesophageal resections is poor to date. There is, moreover, no universal management algorithm due to unique clinical factors impacting therapy such as age, functional status, local expertise, and rates of chyle accumulation. There is a paucity of high-quality prospective studies directly comparing treatment modalities; therefore, approaches are based upon clinical experience and low-quality reports. The improved nomenclature, and the advent of IR approaches, presents an opportunity to advance our understanding and management of this occasionally complex problem across the spectrum of its clinical presentation.

## ETHICS APPROVAL

Not needed, as this study did not involve primary data collection or analysis.

## AVAILABILITY OF DATA AND MATERIAL

Data are fully available from the peer reviewed studies included in this systematic review.

## AUTHORS’ CONTRIBUTIONS

R.P., T.N., and N.E.D. screened and selected articles. P.S. and R.P. extracted data, assessed for bias, and wrote the first draft. N.E.D., J.V.R., and C.L.D. conceived the idea for the study. All authors participated in manuscript preparation, writing and editing.

## Supplementary Material

Supplementary_appendix_doab012
